# Enzyme Immobilization on Metal Organic Frameworks: the Effect of Buffer on the Stability of the Support

**DOI:** 10.1021/acs.langmuir.2c01630

**Published:** 2022-10-26

**Authors:** Kim Shortall, Fernando Otero, Simon Bendl, Tewfik Soulimane, Edmond Magner

**Affiliations:** Department of Chemical Sciences, Bernal Institute, University of Limerick, V94 T9PX Limerick, Ireland

## Abstract

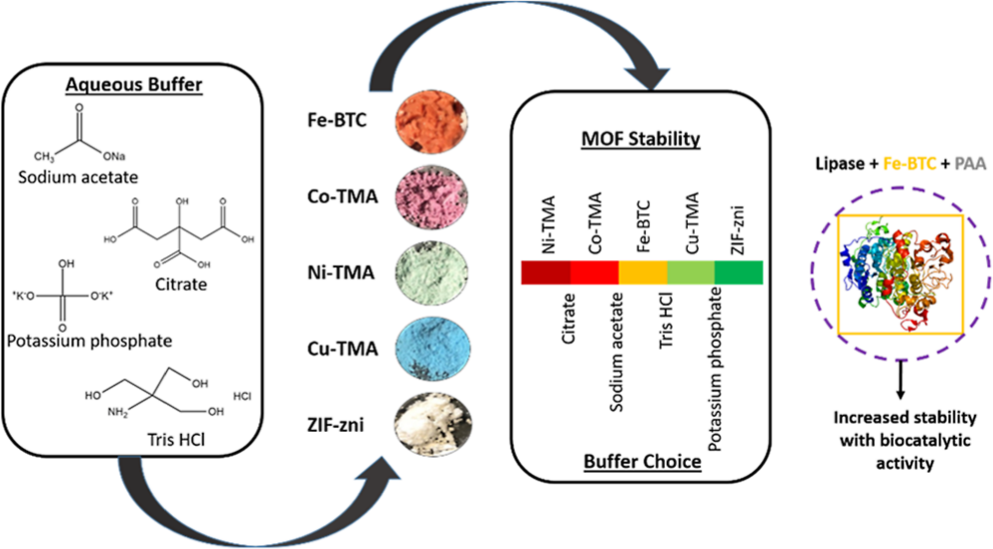

Metal organic frameworks (MOFs) have been used to encapsulate an array of enzymes in a rapid and facile manner; however, the stability of MOFs as supports for enzymes has not been examined in detail. This study examines the stability of MOFs with different compositions (Fe-BTC, Co-TMA, Ni-TMA, Cu-TMA, and ZIF-zni) in buffered solutions commonly used in enzyme immobilization and biocatalysis. Stability was assessed via quantification of the release of metals by inductively coupled plasma optical emission spectroscopy. The buffers used had varied effects on different MOF supports, with incubation of all MOFs in buffers resulting in the release of metal ions to varying extents. Fe-BTC was completely dissolved in citrate, a buffer that has a profound destabilizing effect on all MOFs analyzed, precluding its use with MOFs. MOFs were more stable in acetate, potassium phosphate, and Tris HCl buffers. The results obtained provide a guide for the selection of an appropriate buffer with a particular MOF as a support for the immobilization of an enzyme. In addition, these results identify the requirement to develop methods of improving the stability of MOFs in aqueous solutions. The use of polymer coatings was evaluated with polyacrylic acid (PAA) providing an improved level of stability. Lipase was immobilized in Fe-BTC with PAA coating, resulting in a stable biocatalyst with retention of activity in comparison to the free enzyme.

## Introduction

Metal organic frameworks (MOFs) are highly porous materials^1,2^ composed of a metal ion and an organic linker that are assembled into a three-dimensional structure. The composition and structure of MOFs are enormous due to the numerous permutations available for synthesis of different frameworks using the range of available metals and organic linkers, enabling the tuning of properties such as topology, pore size, surface area, and functionalization.^3^ This scope makes MOFs to be of significant interest for a variety of applications that include gas storage and separation,^4,5^ heterogeneous catalysis,^6,7^ sensing,^8^ and drug delivery.^9^

More recently, MOFs have been applied for the immobilization of enzymes, encapsulating enzymes within the pores via in-situ immobilization. The use of MOFs provides a convenient approach for the encapsulation of enzymes in aqueous solutions. A range of MOFs have been employed for the in-situ encapsulation of enzymes and include Fe-BTC, MAF-7, ZIF-8, and ZIF-90.^10,11^ The conditions used in the synthesis, including aqueous media, mild pH, and ambient temperature, coupled with the rapid formation of MOFs, are highly advantageous for enzymes. Fe-BTC has been used to encapsulate a range of enzymes, including alcohol dehydrogenase (ADH),^11,12^ lipase (Lip),^11,13^ glucose oxidase (GOx),^11^ and laccase (Lac).^13,14^ Encapsulation of enzymes in Fe-BTC provides several advantages. Compared to their free counterparts, the immobilized enzymes possessed higher stability under harsh conditions such as elevated temperatures,^12^ the presence of organic solvents,^12^ and extremes of pH.^11,12^ Enzymes immobilized in Fe-BTC can also be used several times without loss of activity.^11,12,14^

Despite extensive studies on the immobilization of enzymes in MOFs and analysis of the activity of the encapsulated enzyme, the structural and chemical stability of MOFs used as enzyme supports has not been described in detail. A common aspect of enzyme immobilization studies is a focus on enzyme stability within the MOF or enzyme leaching studies rather than the stability of the support itself.^11,15,16^ Such a focus is not just restricted to the use of MOFs, similar comments can be applied to other immobilization supports.^17^ One exception is the use of supports functionalized with metal ions such as Ni^2+^^18,19^ that may be loosely bound and leach readily. This can result in contamination and potential interference with the reaction.^20^ Support stability is a critical consideration because the enzymes can leach out unless the integrity of the composite is maintained upon use or storage in aqueous environments. Additionally, contamination of desired products with either the metal or linker components can occur upon breakdown of the support.^21^

Aqueous environments represent especially challenging conditions for MOFs because of the potential of the hydrolytic cleavage of weak coordination bonds which constitute the MOF backbone.^22^ Reports have described the low hydrolytic stability^21,23,24^ of MOFs, but these aspects are generally not considered in detail when examining enzyme immobilization.^11,25−27^ The stability of MOFs in water is determined by the strength of the metal-linker coordination bonds^28^ and the level of saturation of metal sites,^23^ resulting in different MOFs displaying varying hydrolytic stability. Additionally, the composition of buffers commonly used in enzymatic reactions needs to be considered as the buffer can affect the MOF’s structural integrity, in particular, by disturbing the coordination bonds.^21,29,30^ Breakdown of the MOF structure in buffered solutions^29,31,32^ is a key challenge facing the use of MOFs for the immobilization of enzymes, as buffers are required for enzymatic activity. As an example, UiO-66 was found to rapidly degrade in both *N*-ethylmorpholine and phosphate buffers, while 4-(2-hydroxyethyl)-1-piperazineethanesulfonic acid (HEPES) was found to be the most benign buffer, and low concentrations of 2-amino-2-hydroxymethyl-propane-1,3-diol (Tris) were tolerable.^21^ Similarly, ZrMOF and MIL-101(Fe) decomposed rapidly in phosphate buffered saline (PBS).^31^ ZIF-8 was also unstable in many biologically relevant solutions such as citrate and 2-(*N*-morpholino)ethanesulfonic acid (MES), while Tris and HEPES allowed for higher stability.^29^ This indicates that different MOFs possess varying levels of stability in different buffers. Structural degradation and subsequent leaching of the enzyme were demonstrated in buffer solutions used for the immobilization of GOx and alkaline phosphatase in ZIF-8.^29^ Buffer salts that contained a number of carboxylic acid groups and lower p*K*_a_ values decreased the stability of the ZIF-8 enzyme composite. Acetate buffer had a limited effect on the stability of the composites, malate buffer had a moderate effect, and in citrate buffer, the MOF was completely solubilized, releasing all of the encapsulated enzyme.^29^ Additionally, MES and sodium phosphate buffer had detrimental effects on the stability of ZIF-8, while Tris buffer had little destabilizing effects.

The breakdown of MOF supports can be linked to the degree of complexation of buffer components with the divalent cations in the structure, as well as the degree of coordinative saturation of the metal ion in the MOF structure. As the MOFs (different metals and the degree of their bond saturation) and the buffers used in immobilized enzymatic systems can vary, this underlines the need to examine MOF–enzyme–buffer systems to ensure that optimal support stability can be achieved in the biocatalytic system. As previously demonstrated, MOFs display a range of stability in different buffers, an attribute that must be examined when using MOFs as supports. Studies have also focused on increasing the hydrolytic stability of MOFs via increasing their hydrophobicity^33,34^ or by enhancing the strength of the metal–ligand bond. Studies used Zr rather than Cu or Zn^35^ when the stability of Cu- or Zn-MOF materials was unfavorable for desired applications.

In this study, we have examined the effects of a range of biologically relevant buffers on the stability of a selection of MOF supports. We have identified buffer compositions which are compatible with or detrimental to the supports. Five MOFs were analyzed and composed of different metal nodes (Fe, Co, Ni, Cu, and Zn) and organic linkers of trimesic acid (Fe-BTC, Co, Ni, and Cu-TMA) or imidazole (ZIF-zni). The stability of the support was assessed by analyzing the concentration of the free metal component after exposure to buffer at an ambient temperature. On storage in buffer for 24 h, all MOFs displayed some release of metal. Potassium phosphate and Tris buffers were the most compatible, while citrate buffer had very detrimental effects on MOFs’ structural stability. Sodium acetate was identified as a suitable buffer for use at an acidic pH as significantly less degradation of the support was observed. The most stable MOFs—Fe-BTC, Cu-TMA, and ZIF-zni—were successfully used to encapsulate two dehydrogenase enzymes—aldehyde dehydrogenase (ALDH_Tt_)^36^ and lactate dehydrogenase (LDH). Additionally, we describe the screening of a range of polymers to enhance the stability of MOFs in buffer. Polyacrylic acid (PAA) provided the most improved stability while also maintaining the enzymatic activity of Lip@MOF.

## Experimental Section

Cobalt chloride hexahydrate (CoCl_2_·6H_2_O, 98–102%) and copper (II) sulfate pentahydrate (Cu(II)SO_4_·5H_2_O, 99%) were purchased from Fisher Scientific (Ireland). All other reagents were purchased from Sigma Aldrich at ≥99% purity, apart from iron chloride hexahydrate (FeCl_3_·6H_2_O, 97%), zinc nitrate hexahydrate (Zn(NO_3_)_2_·6H_2_O, 98%), sodium hydroxide (NaOH >95%), trimesic acid (H_3_BTC >95%), hexanal (98%), ethanol (96%), potassium phosphate dibasic (K_2_HPO_4_, ≥98%), hydrochloric acid (HCl, 37%), nitric acid (HNO_3_, 70%), l-lactic dehydrogenase from rabbit muscle (LDH, 800–1200 U/mg), lipase B Candida antarctica, recombinant from *Aspergillus oryzae* (Lip, ∼9 U/mg), polyethylene glycol (PEG, average molecule weight 3400), PAA (average M.W. ca. 2000), and polyethylenimine (PEI) (low molecular weight), which were purchased from Sigma Aldrich (Ireland). All reagents were used as received without further purification. Deionized water (18.2 MΩ cm) was used for the preparation of all aqueous solutions.

### Synthesis of Fe-BTC

Fe-BTC was synthesized as previously described.^11^ Solution 1 was prepared by dissolving 0.269 g of trimesic acid (H_3_BTC) in 3.6 mL of 1.06 M NaOH resulting in a final pH of 6–8; solution 2 consisted of 6.388 mL of deionized water; and solution 3 was prepared by dissolving 0.508 g of FeCl_3_·6H_2_O in 10 mL of H_2_O. Solution 2 was added to solution 1, and solution 3 was then added dropwise into the mixture under gentle magnetic stirring. This procedure resulted in the immediate appearance of a reddish brown solid (Figure S1). The resultant suspension was maintained under stirring at room temperature for 10 min. The obtained solid was recovered by vacuum filtration, washed with deionized water (approx. 30 mL in 3 washes), and dried at room temperature for approx. 30 min. The resulting Fe-BTC was stored at 4 °C under dry conditions using silica gel.

### Synthesis of Co-TMA, Ni-TMA, and Cu-TMA

Trimesic acid-based MOFs with different metal nodes (Co, Ni, and Cu) were prepared as previously reported^37^ and are denoted as Co-TMA, Ni-TMA, and Cu-TMA. An aqueous solution of 95 mM trimesic acid was prepared by dissolving 1 g of trimesic acid in 50 mL of H_2_O. The organic linker solution was adjusted to pH 7 by dropwise addition of 1 M NaOH until all of the trimesic acid was dissolved. Metal ion solutions of CoCl_2_, NiCl_2_, or Cu(II)SO_4_ were prepared in water at a concentration of 75 mM. The appropriate metal ion solution (50 mL) was then added dropwise into the trimesic acid solution in a beaker under constant stirring at a rate of ca. 1000 rpm. After approximately 10 min of stirring, depending on the nature of the metal ion, different colored precipitates, for example, pink for Co(II), light green for Ni(II), and blue for Cu(II), were observed upon complexing with trimesic acid (Figure S2). Mixing continued for approx. 17 h. Subsequently, the obtained MOFs were filtered using filter paper under vacuum and washed with excess water, 10 mL of ethanol, and again with water. The MOF was then dried at room temperature.

### Synthesis of ZIF-zni

ZIF-zni was synthesized as previously reported.^38^ 2 mL of 0.1 M sodium acetate solution pH 5 was added to a solution of imidazole (0.851 g of imidazole in 10 mL of water). 1 mL of 3.1 M Zn(NO_3_)_2_ was added dropwise to the imidazole solution with gentle magnetic stirring, resulting in the appearance of a white precipitate. The suspension was stirred at room temperature for 45 min. The ZIF-zni was collected via vacuum filtration, washed with water (approx. 30 mL in 3 washes), and dried for 3 min. The obtained ZIF-zni solid (Figure S3) was stored at 4 °C in dry conditions using silica gel.

### Scanning Electron Microscopy Characterization of MOFs

A scanning electron microscope (SEM, Hitachi SU-70) was used to determine the morphology of the MOFs. SEM images were taken at two different magnifications: 0.35 and 1.0k at a working distance of 11.6–12.3 mm and at an accelerating voltage of 10 kV. The average length of the Co- and Ni-TMA MOFs was determined using ImageJ software on the SEM images at 1.0 kV, using at least 10 measurements.

### Storage Stability of MOFs in Various Buffers

Inductively coupled plasma optical emission spectroscopy (ICP-OES) was used for quantification of metals in solution using an Agilent 5100 ICP-OES equipped with an Agilent SPS 4 autosampler. A standard curve of Fe, Co, Ni, Cu, and Zn was prepared in the range of 0.5–200 ppm in 1 M nitric acid.

10 mg/mL of ZIF-zni was incubated in 1 M nitric acid for 24 h to allow for complete degradation of the MOF and to ensure that all of the zinc present had dissolved. Trimesic acid-based MOFs were incubated in 100 mM citrate buffer at pH 5 and concentrations of 10 mg/mL (Fe-BTC) and 5 mg/mL (Co-TMA, Ni-TMA, and Cu-TMA) for 24 h in 100 mM citrate buffer at pH 5. The solutions were then diluted with 1 M nitric acid and filtered through a 0.2 μm PTFE filter prior to ICP-OES analysis.

The storage stability of Fe-BTC, Co-TMA, Ni-TMA, Cu-TMA, and ZIF-zni was analyzed in a range of buffers that are commonly used in enzyme assays. 10 mg/mL of MOF was incubated in 10 mM buffer at pH 5 (citrate or sodium acetate), pH 7 (potassium phosphate), and pH 9 (Tris–HCl) at room temperature for a period of 24 h (unless otherwise stated). ICP-OES was used to determine the amount of metal released during storage.

### Production of ALDH_Tt_

Aldehyde dehydrogenase from *Thermus thermophilus* (ALDH_Tt_) was expressed in *E. coli* BL21(DE3) and subsequently purified as previously reported.^36^

### In-Situ Immobilization of ALDH_Tt_ in Fe-BTC

Enzyme immobilization in Fe-BTC was carried out as above with the addition of the appropriate amount of enzyme to solution 2. The enzyme encapsulation efficiency was calculated with the Bradford assay on the MOF supernatant. ALDH_Tt_ immobilized in Fe-BTC is denoted as ALDH_Tt_@MOF.

### Characterization and Enzymatic Activity of ALDH_Tt_@MOF

The presence of ALDH_Tt_ in Fe-BTC was determined using sodium dodecyl-sulfate polyacrylamide gel electrophoresis (SDS-PAGE) analysis. A sample of ALDH_Tt_@MOF was crushed to a fine powder using a mortar and pestle. 750 μL of 4× Laemmli SDS buffer was added to the MOF sample and boiled at 100 °C for 10 min. The mixture was then centrifuged at 8000×*g* for 5 min, and the supernatant was collected. Similarly, 450 μL of Fe-BTC supernatant was added to 150 μl of 4× Laemmli SDS buffer and boiled. 15 μL of each sample was then run on a 12% SDS-PAGE for analysis.

The catalytic activity of ALDH_Tt_@MOF was determined by measuring the increase in absorbance at 340 nm due to the production of NADH by the enzyme using a Cary 60 UV–vis spectrophotometer equipped with a temperature controller. The activity of ALDH_Tt_@MOF was measured at 25 °C in 10 mM potassium phosphate at pH 8 unless otherwise stated. The reaction mixture consisted of 0.1 mL of 10 mg/mL ALDH_Tt_@MOF in buffer, 2 mM NAD^+^, 10 mM potassium phosphate at pH 8 and 2 mM hexanal in a final reaction volume of 1.8 mL. All ALDH_Tt_@MOF samples required stirring during spectrophotometric analysis at 260 rpm to maintain a homogeneous suspension.

### Stability Study of Fe-BTC under Enzymatic Reactor Conditions

Fe-BTC was subjected to enzyme assay conditions by combining 1.7 mL of 100 mM buffer (citrate buffer pH 5, potassium phosphate buffer pH 6 and 7, Tris–HCl pH 9) and 100 μL of 10 mg/mL MOF-buffer suspension with stirring at 260 rpm while monitoring absorbance at 340 nm for 10 min.

### Immobilization of LDH in ZIF-zni and Cu-TMA

2 μL of LDH (11.1 mg/mL, ∼800 U/mg) was added to 2 mL of 0.1 M acetate buffer at pH 5, and immobilization in ZIF-zni was carried out as in section synthesis of Zif-zni. This preparation is denoted as LDH@ZIF-zni.

The Cu-TMA protocol outlined was adapted for enzyme immobilization. 4 μL of LDH (11.1 mg/mL, ∼800 U/mg) was added to 10 mL of 95 mM trimesic acid, pH 7. 10 mL of 75 mM Cu(II)SO_4_ was added dropwise to the 10 mL trimesic acid—enzyme solution under gentle magnetic stirring (∼200 rpm) and kept under stirring for 30 min. The resultant solid was collected via vacuum filtration, washed with H_2_O (30 mL in three washes), and dried for 30 min. This preparation is denoted as LDH@Cu-TMA.

The enzymatic activity of LDH, LDH@ZIF-zni, and LDH@Cu-TMA was monitored spectrophotometrically at 340 nm at 37 °C for 5 min using a Cary 60 UV–vis spectrophotometer equipped with a temperature controller. The assay solution consisted of 0.12 mM NADH and 2.3 mM sodium pyruvate in 100 mM sodium phosphate buffer at pH 7.5 and either 100 μL of LDH (0.5 U/mL stock) or 200 μL of 10 mg/mL of LDH@ZIF-zni and LDH@Cu-TMA suspension.

### Testing of Additives for Increased MOF Stability in Aqueous Solutions

Fe-BTC was incubated for 24 h at room temperature in a 10 mg/mL solution of 10 mM citrate at pH 5 supplemented with different concentrations of the salts (0.5 and 1 M NaCl and 0.2 and 0.5 M imidazole). 10 mg/mL of Fe-BTC was incubated in a mixture of 10 mM citrate at pH 5, supplemented with 4 and 8% v/v of various polymers, PAA (stock 30% wt solution), PEG (stock 30% wt solution), PEI, or polysorbate Tween 20 for 24 h at ambient temperature. The release of Fe^3+^ over a 24 h period was quantified by measuring the absorbance at 295 nm (Figure S4).

The enzymatic activities of ALDH_Tt_, LDH, and Lip were analyzed in the presence of 4 and 8% v/v PAA. The activities of ALDH_Tt_ and LDH were analyzed as described above with the addition of PAA. Lip activity was analyzed by monitoring the increase in absorbance at 348 nm at 25 °C for 5 min using a molar extinction coefficient of 5300 M^–1^ cm^–1^ (Figure S5). Previous reports^11,39^ use ε = 5150 M^–1^ cm^–1^; however no experimental data is shown. The assay solution consisted of 0.4 mM *p*-nitrophenyl acetate and 50 μL of Lip (0.056 mg/mL), with supplementation of 4 and 8% v/v PAA when required.

Lip was immobilized in Fe-BTC^11^ as described above with the addition of 1.5 mg/mL to solution 2. Enzymatic activity was monitored at 348 nm at 25 °C for 2 min with stirring at 260 rpm. Addition of 200 μL of 10 mg/mL Lip@MOF suspension to 1.9 mL of 0.4 mM *p*-nitrophenyl acetate (*p*-NPA), supplemented with 4 and 8% PAA when required, was performed for activity analysis.

## Results and Discussion

Screening of a selection of MOFs was carried out to identify stable supports. All of the MOFs chosen could be synthesized rapidly in aqueous media at room temperature, making them suitable candidates for in situ immobilization. Four trimesic acid-based MOFs were explored, which were composed of different metal nodes—Fe-BTC, Co-TMA, Ni-TMA, and Cu-TMA.^11,37^ Furthermore, an imidazole-based MOF utilizing Zn^2+^, ZIF-zni, was investigated^38,40^ to examine the effects of different organic linkers and degrees of metal saturation on stability. The majority of MOFs suffer from low structural stability and particularly low hydrolytic stability when stored in a humid environment.^22,23,41,42^ Widely studied carboxylate ligand MOFs such as MOF-5 and MOF-177 are water sensitive,^43,44^ while some zeolite-based MOFs (ZIFs) are resistant to hydrolysis.^34^

SEM images of each MOF demonstrated different surface morphologies. Co- and Ni-TMA MOFs possessed rod-like features, with an average length of 11.4 ± 3.0 and 7.9 ± 1.6 μm, respectively. Fe-BTC, Cu-TMA, and ZIF-zni displayed a characteristic nanoflower morphology (Figure S6). The metal ion content of the MOF was quantified via complete degradation of the MOF in solution with subsequent analysis by ICP-OES (Table S1). ZIF-zni was fully dissolved after overnight immersion in 1 M nitric acid. Trimesic acid-based MOFs were more stable in 1 M and in concentrated (70%) nitric acid, with the majority of the MOF solid still visible after overnight incubation. In 100 mM citrate buffer at pH 5, these MOFs dissolved completely after 24 h. The structure of amorphous Fe-BTC was, until recently, poorly understood.^13,45^ It was often compared to its crystalline counterpart MIL-100(Fe),^46^ which is formed by trimers of iron octahedra linked by trimesic acid. Recently significant advancement^45^ into the understanding of the structure of Fe-BTC demonstrated that it displays a degree of order between an extended crystal and an entirely amorphous solid, possessing a nanocomposite structure. No free, unincorporated iron trimers (FeO_6_ octahedra that cluster around a shared oxo-anion unit) are present in MIL-100(Fe), and all Fe^3+^ trimers are present within the tetrahedral assemblies formed by trimesic acid linkers.^46^ Fe-BTC contains a considerable proportion of free trimers (36–61%), contributing to its semi-crystalline nature.^45^ Given that the synthesis of Fe-BTC occurs within seconds of mixing, it is likely that cross-linking of trimer units occurs in a disordered fashion via the organic linkers. These compete with the tetrahedral assembly process, resulting in the presence of both features in the final structure.^45^ In this work, a range of mass metal % was calculated for Fe-BTC, dependent on whether six or three individual trimesic acid molecules form a coordination bond with the octahedral Fe^3+^. This resulted in the theoretical mass % of Fe^3+^ in Fe-BTC being in the range of 4.2–8.1%. Using ICP-OES, the quantity of Fe^3+^ was determined to be 3.4%.

The structures of the other investigated trimesic acid-based MOFs—Co, Ni, and Cu-TMA—have been previously reported.^37^ Their structure contains only two coordination bonds formed with the metal ion, resulting in the presence of unsaturated metal ions. The chemical formula of these MOFs is M(II)_3_BTC_2_. The theoretical metal content of Co-TMA, Ni-TMA, and Cu-TMA was 29.6, 29.5, and 31.2%, respectively, in agreement with the content determined experimentally (Table S1). The ZIF-zni structure is described as crystalline and tetragonal in nature.^40,47^ The saturated tetrahedral metal center of Zn^2+^ linked by imidazole, Zn(Im)_2_, forms ZIF-zni. The theoretical metal mass % is 32.4%, with an amount of 21.0% determined experimentally. For all MOFs analyzed, the experimental determination of metal content was lower than the theoretical value, perhaps indicative of a small degree of unspecific binding of linkers to unsaturated metal ions.

**Figure 1:**
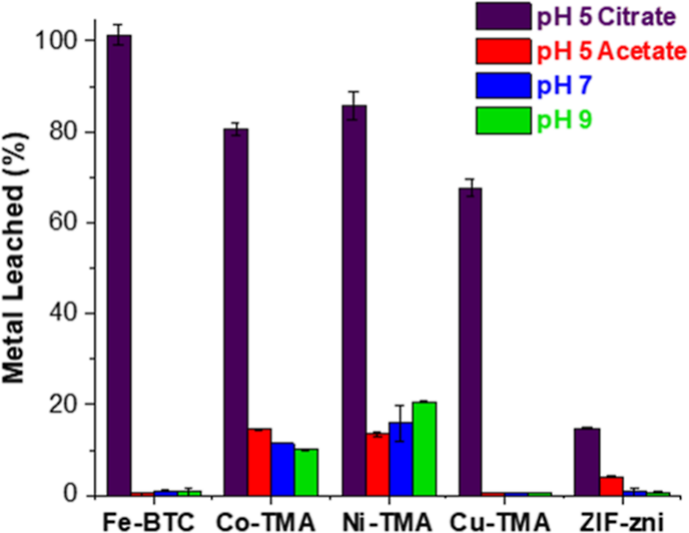
Storage stability of a selection of MOFs in 10 mM buffer (citrate and acetate pH 5, potassium phosphate pH 7, and Tris–HCl pH 9) over 24 h, demonstrating % of metal ions leached with respect to total metal content. Determined by ICP-OES analysis. The error bars refer to different experiments.

Following storage in various buffers for 24 h, release of metal ions from all MOFs occurred, with the amounts dependent on the buffer utilized (Figure 1). An early study^30^ investigated the metal-buffer binding constants of a range of 14 buffers for complexing with metals. It was demonstrated that the level of complexation varied with different buffer-metal combinations. Generally, Cu^2+^ had the highest binding constant compared to Ca^2+^, Mg^2+^, and Mn^2+^ across buffers. Here, a significant increase in instability was observed in citrate buffer (pH 5) for all MOFs analyzed. ZIF-zni possessed the highest degree of stability in citrate buffer (pH 5), with 14.8% of Zn^2+^ leached. Higher levels (68–100%) of metal ions were released with trimesic acid-based MOFs. Co, Ni, and Cu-TMA possess just two occupied coordination sites,^37^ leaving binding sites free. Interactions between the chelating citrate^48−51^ and unsaturated metal sites may result in dissociation of the metal ion and breakdown of the MOF structure.^48−50^ The tetrahedral Zn^2+^ center in ZIF-zni is saturated through coordination with four imidazole molecules, inhibiting the action of citrate. In addition, it has been highlighted that a major disadvantage of MOFs containing coordinatively unsaturated sites is their hydrolytic instability.^23^ A study on Cu-TMA showed that water molecules displaced organic linkers from the copper node, resulting in the irreversible decomposition of the framework.^23,52^

All MOFs demonstrated higher stability in sodium acetate buffer at a pH of 5 with respect to citrate buffer, as previously demonstrated using ZIF-8.^29^ Fe-BTC and Cu-TMA released less than 1% of metal ions in acetate buffer. This indicates that the interaction between the citrate ion and the MOFs is the determining factor, rather than the pH. Due to its chelating nature,^48,50^ citrate has previously been shown to facilitate breakdown of MOFs. Co-TMA and Ni-TMA had the highest levels of release of metal ions across the buffer range analyzed, rendering them unsuitable for use as support materials in the immobilization of enzymes. Overall, the most stable MOF in this study was ZIF-zni, a material that is considered to be the most stable of ZIF MOFs with good mechanical properties.^53^

Previously, studies have shown that smaller particle size can lead to decreased MOF stability and higher rates of degradation in buffer.^32,54^ However, studies tend to focus on only one MOF, as comparison across supports of different composition, structure, and particle size can be difficult. The MOFs represented here range in size from 150 nm to 11.4 μm (Fe-BTC: 150 nm,^11^ Cu-TMA 1 μm,^55^ ZIF-zni: 3–4 μm,^56,57^ Co-TMA: 7.9 μm, Ni-TMA: 11.4 μm^37^). The stability generally decreased upon increasing particle size, but this is only one of a number of other contributing factors. The rod-shaped MOFs were also the least stable of those examined, while the nanoflower composition was more tolerant to buffer exposure.

**Figure 2:**
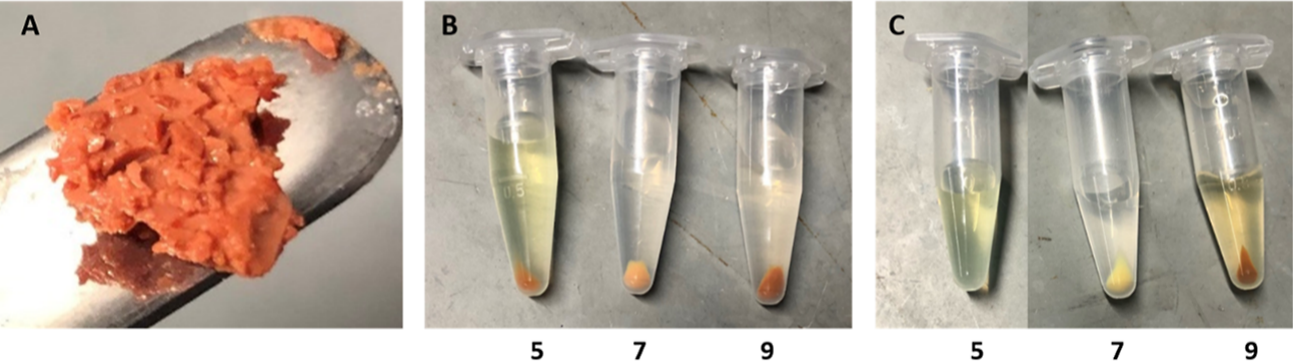
Visualization of Fe-BTC storage samples in citrate pH 5, potassium phosphate pH 7, and Tris–HCl pH 9. (A) Fe-BTC before storage. Fe-BTC samples following 24 h of shaking incubation in (B) 10 mM buffer and (C) 100 mM buffer. The pH of the buffers is listed.

The effect of the buffering capacity was investigated using Fe-BTC. The ability of the buffer to maintain pH upon breakdown of the MOF and the effect of increased buffer strength on support stability were analyzed. Fe-BTC was incubated in 10 and 100 mM buffer (citrate pH 5, potassium phosphate pH 7, and Tris–HCl pH 9). The 100 mM buffered samples maintained the pH following 48 h of incubation. For the 10 mM samples, significant decreases in pH occurred (Table S2). This highlighted that a higher buffer concentration is required to maintain the pH; however, increased buffer capacity has previously been shown to contribute to increased MOF instability.^54^ A recent study^21^ investigated the effects of increasing concentrations (0.01–1 M) of Tris and HEPES buffers on the release of the Ui-066 terephthalate linker. Upon increasing the concentration of buffer, significantly higher amounts of linkers were released. For example, in Tris buffer at pH 7.5, the amount released increased from 10% (0.01 M) to 100% (1 M). Figure 2 displays the pellets obtained via centrifugation following storage of Fe-BTC in solutions of 10 and 100 mM buffer. The 100 mM buffer was more destructive, disrupting the MOF color and, in some cases, the solid phase (no pellet obtained in citrate, pH 5). This confirmed that an increased buffering capacity caused increased structural instability. Incubation of all samples in 10 mM buffer resulted in the recovery of a pellet following centrifugation. Additionally, the 100 mM sample supernatant displayed an orange hue, indicative of higher levels of released Fe^3+^. This data also demonstrates that use of enzyme@MOF composites in lower strength buffers is overall more favorable. Increasing the ionic strength of the buffer also did not allow for increased support stability (discussed below).

From this analysis, we present a selection of MOFs that can be chosen in specific scenarios depending on buffer requirements of the enzyme to ensure concomitant enzyme and support stability. However, it is apparent that the Co-TMA and Ni-TMA are highly unsuitable for biocatalytic applications. Cu-TMA showed promising results with less than 0.5% of Cu released over 24 h in acetate, potassium phosphate, and Tris HCl at pH 5, 7, and 9, respectively. Commercially available Basolite C 300, an analogue of Cu-TMA, demonstrated significant degradation after 3 days of exposure (200 mg, dry, in a 20 mL vial) to 90% relative humidity at room temperature and 40 °C.^58^ In comparison, MIL-100/101(Fe) progressively degraded in water after a few hours.^59^ A recent study demonstrated that the stability of the M(II)_3_(BTC)_2_ MOFs followed a predicted hydrolytic stability trend of Cu-TMA > Co-TMA > Ni-TMA,^23^ consistent with the results in Figure 1. The Cu-TMA structure was reported to remain intact for up to 10 h in water at room temperature. The Co-TMA and Ni-TMA structures collapsed after 1 h.^23^

The above results demonstrated that Fe-BTC, Cu-TMA, and ZIF-zni are suitable for use as supports for the encapsulation of enzymes in solutions of acetate, potassium phosphate, or Tris–HCl but not in citrate buffer. These MOFs were then used to encapsulate two dehydrogenase enzymes, the thermophilic ALDH_Tt_ and LDH, from rabbit muscle. Fe-BTC has previously been used for the immobilization of enzymes, including ADH, Lip, GOx, and Lac, reporting improved catalytic performance in terms of thermal and pH stability.^11,12^ The use of Fe-BTC as a support allows for a rapid and facile in-situ immobilization process for the development of an immobilized enzyme, with times as short as 1 h. Here, we confirm the successful immobilization of ALDH_Tt_, a thermophilic enzyme that can be used at temperatures up to 50 °C with a range of aldehyde substrates including hexanal and terephthalaldehyde^36^ to produce the respective carboxylic acids using NAD^+^ as a cofactor. Three concentrations of enzyme were examined—0.47, 1.80, and 2.34 mg/mL (in aqueous solution 2). Low levels of enzyme were detected in the supernatant (537.5, 192.9, and 0.0 μg, respectively), resulting in high levels of enzyme encapsulation (83–100%). When compared to previous reports, 100% encapsulation of ADH and GOx was achieved, but the amount of Lip encapsulated ranged from 35 to 95%.^11^ Encapsulation of ALDH_Tt_ in Fe-BTC was confirmed by SDS-PAGE (Figure S7).^13,60^ The presence of the 59 kDa ALDH_Tt_^36^ was confirmed in the analyzed Fe-BTC samples, while no protein was detected in the supernatant.

**Figure 3:**
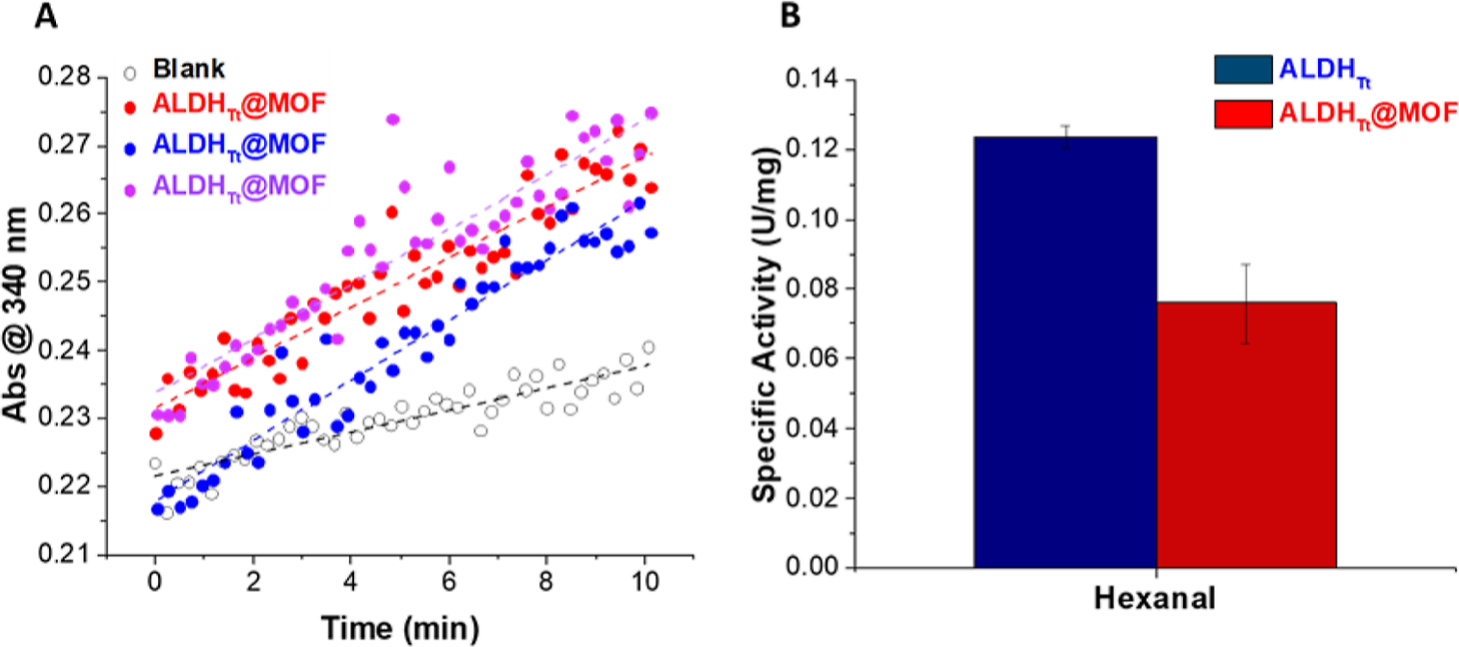
Activity of ALDH_Tt_@MOF using hexanal at 25 °C. (A) Plot of absorbance vs time for blank (Fe-BTC) and ALDH_Tt_@MOF samples following the production of NADH by the enzyme at 340 nm. (B) Comparison of activity of soluble ALDH_Tt_ and ALDH_Tt_@MOF. The error bars refer to different measurements.

The immobilized enzyme retained approx. 60% of activity in comparison to the free enzyme using hexanal as the substrate (Figure 3). This reduction in activity could be due to activity loss during the immobilization process or possible diffusion limitations. The caged MOF structure may act as a barrier to the relatively bulky cofactor NAD^+^. The catalytic activity of ADH@MOF from *Saccharomyces cerevisiae*(11) decreased significantly due to NAD^+^ diffusion limitations into the Fe-BTC.

In running a control experiment, a response was obtained for a blank sample containing Fe-BTC with no enzyme under the same reaction conditions (Figure 3). This raised questions about the stability of the support during the assay and the potential leaching of Fe^3+^ during analysis. The stability of Fe-BTC under the assay conditions was investigated.

**Figure 4:**
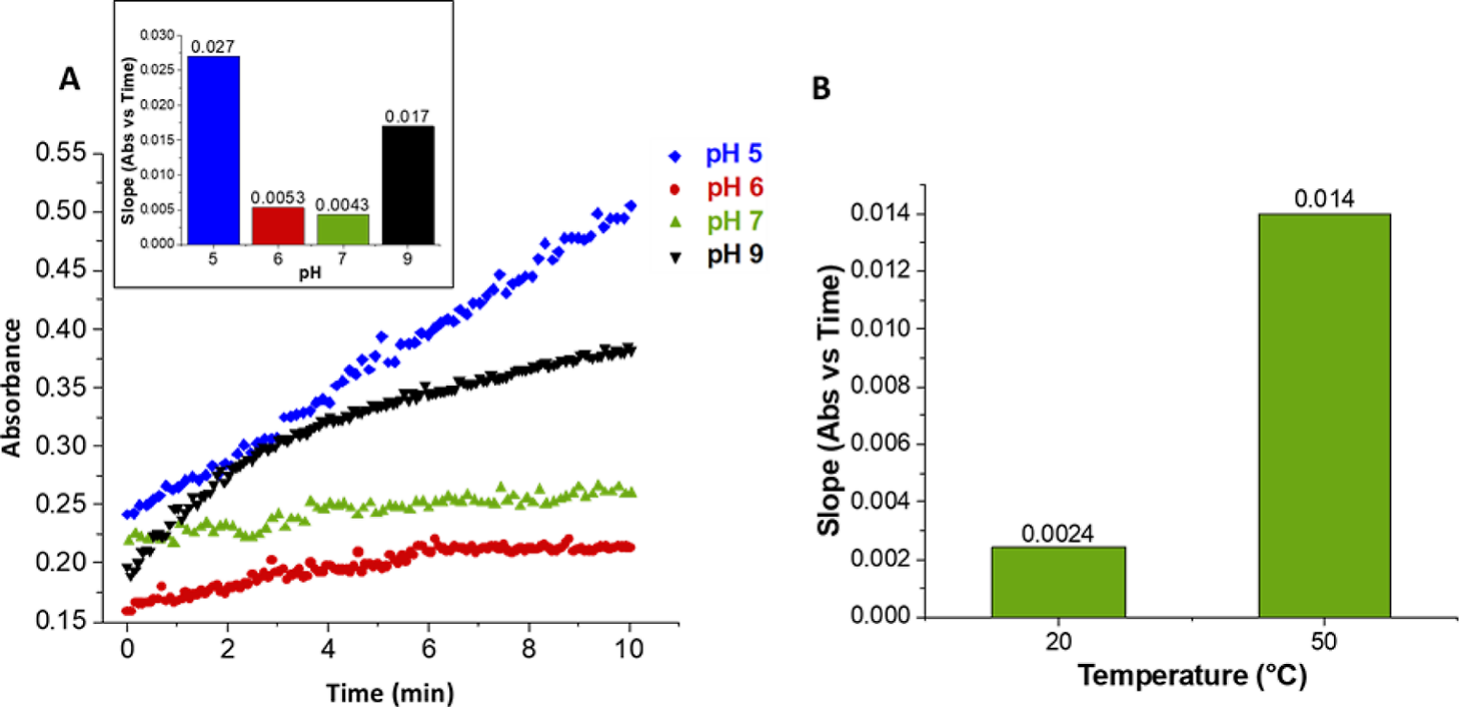
Stability of Fe-BTC under enzymatic assaying conditions in 100 mM buffer monitored at 340 nm (A) varying pH at 25 °C. The inset demonstrates the associated slopes. (B) Varying temperatures in 100 mM potassium phosphate, pH 7.

While Fe-BTC demonstrated numerous advantages for enzyme immobilization, the support is hampered by its instability both during storage and upon assaying for short periods of time. For enzymes such as dehydrogenases, monitoring the appearance or disappearance of NADH at 340 nm is difficult. Greatly limited detection abilities via UV–vis spectrometry for enzymes immobilized in Fe-BTC have been identified. Assaying of Fe-BTC resulted in an increase in absorbance over time at 340 nm and varied depending on the buffer composition and temperature (Figure 4). A significantly increased change in absorbance over time was observed at pH 5, 6.3-fold higher than that at pH 7, consistent with the storage stability data above. The lowest response was seen at pH 7, with an increase of approx. fourfold at pH 9 in Tris–HCl. As the ALDH_Tt_ is thermophilic, it was desirable to use the immobilized biocatalyst at 50 °C. However, use of the support at elevated temperatures demonstrated added difficulties. Fe-BTC displayed a 5.8-fold higher increase in absorbance at 50 °C compared to 20 °C at pH 7 (Figure 4), demonstrating that the MOF was unsuitable as a support under these conditions. Temperature and pH use in conjunction with Fe-BTC is therefore greatly limited to neutral conditions at mesophilic temperatures. The use of UV–vis analysis at wavelengths above 400 nm is feasible with no interference occurring. Use in conjunction with colorimetric detection or enzyme-coupled assays is feasible, but the stability of MOF supports needs to be closely monitored. Previously, immobilization of GOx within the Fe-BTC demonstrated 2.4 times higher activity than that of the free enzyme,^11^ for detection of ABTS at 720 nm through use of horse radish peroxidase (HRP). We analyzed the response of Fe-BTC under assaying conditions at pH 5, 7, and 9 at 720 nm. No increase in absorbance was detected over time.

Additionally, Cu-TMA and ZIF-zni were examined for the immobilization of the enzyme LDH. However, the activity of LDH was greatly diminished upon immobilization in ZIF-zni (residual activity of 6.5%) and demonstrated no detectable activity in Cu-TMA (Figure S8). This indicates that encapsulation of dehydrogenases in MOFs is not feasible, as was previously described with ADH immobilized in Fe-BTC, demonstrating 6% residual activity compared to the soluble enzyme.^11^

The stability of the supports can potentially be addressed by post-synthetic modification. Surface coating is a simple, direct, and effective method that has been used to increase the stability of MOFs.^61^ A range of materials were examined to bind free coordination sites on MOFs and increase the stability of the support and/or coat the outer surface, preventing the ingress of water. The addition of NaCl, imidazole, and a selection of polymers (PEG, PEI, PAA, and Tween 20) to Fe-BTC storage in 10 mM citrate at pH 5 was investigated. The stability of the MOF in the presence of additives was determined by visual analysis and via monitoring the absorbance at 295 nm following incubation at ambient temperature for 24 h. The addition of NaCl (0.5 and 1 M) resulted in no visible increase in stability but with the deposition of salt on the MOF surface. Similarly, the addition of imidazole did not increase stability. Polymer coating of MOFs has been previously demonstrated to shield the surface of the MOF and to improve stability in aqueous buffered solutions.^31,62,63^ MIL-101(Fe) and ZrMOF degraded rapidly in PBS buffer. Upon addition of PAA and poly(*N*,*N*′-bis(acryloyl)cystamine) (PAC), the MOFs were stable after 15 h as shown by TEM.^31^ Similarly, ZIF-8 modified with PEG had increased stability in acetate buffer from instant dissolution to remaining stable for more than 3 h.^31^ ZrMOF-PAA also possessed increased stability. The absorbance at 420 nm was monitored for the release of the organic linker (meso-tetra(4-carboxyphenyl) porphine) from the MOF structure. The amount of linker released from ZrMOF (in 1× PBS) was 17.8% after 1 h, compared with a value of 4.5% from ZrMOF-PAA.

**Figure 5:**
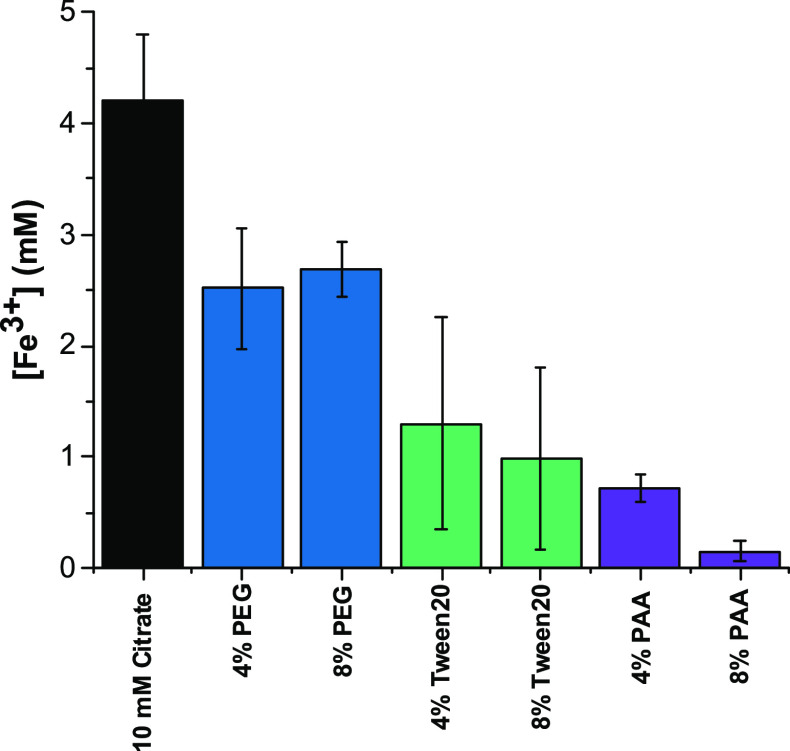
Enhanced stability of Fe-BTC by the addition of polymers to the storage solution of 10 mM citrate at pH 5. Quantification of Fe^3+^ release after 24 h of storage for control and polymer samples of 4 and 8% v/v for demonstration of stabilizing effects of each polymer. The error bars refer to different experiments.

Polymers with different properties (Table S3),^64^ were examined for their ability to stabilize Fe-BTC via simple addition to the storage solution. Concentrations of 4 and 8% v/v were investigated. PAA conferred the best stabilizing effect of the polymers selected. The resulting solution was visibly clearer (no yellow tinge) than when PAA was omitted (Figure S9). This was further confirmed by UV–vis analysis, with significantly less Fe^3+^ released than when no polymer was added (Figure 5). A higher concentration of PAA also conferred higher stability. 8% PAA resulted in 3.6% release compared to the control, whereas 4% PAA demonstrated 17.3%. This may have been due to the structure of PAA containing accessible carboxylate groups, which could interact with free metal ions in the support to develop a polymer coating. This would block the access of citrate for complexation with the metal. Previously, ionic polymers have been shown to form coatings on the outer surface of MOFs^64^ and could contribute to their stability. PAA showed the highest degree of complexation with Zr-fum MOF, compared to polyamidoamine and PEI. PAA has also been co-encapsulated with an enzyme (HRP) in ZIF-L and allowed for increased support structural stability.^65^ However, in-situ immobilization of PAA in Fe-BTC was unachievable as PAA precipitated upon interaction with trimesic acid. Tween 20 proved suitable for increasing Fe-BTC stability (Figures 5 and S9), while PEG displayed slightly increased stabilizing effects. PEI resulted in a highly unfavorable interaction and should be avoided (Figure S9).

**Figure 6:**
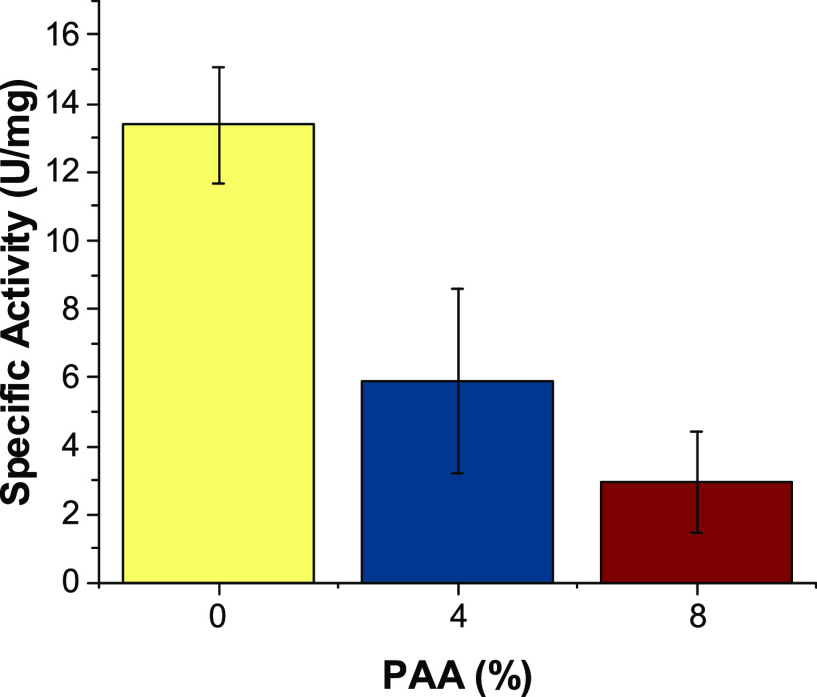
Lip@MOF activity using *p*-NPA on the addition of PAA at different concentrations. The error bars refer to different measurements.

While the use of polymers, particularly PAA, allowed increased Fe-BTC stability during storage, the effect of PAA on enzyme activity is important. Enzyme activity in solution and in MOF was then investigated on the addition of PAA. The activities of ALDH_Tt_, LDH, and Lip in solution were examined after the addition of 4 and 8% PAA. The enzymes remained active with the exception of LDH at 8% v/v PAA (Figure S10). Lip was the most tolerant of PAA at both concentrations (69.6 and 37.3% residual activity). Its activity, when immobilized in Fe-BTC,^11^ resulted in a similar activity trend to that of soluble Lip (Figure S11). With increasing PAA concentration, the activity of both Lip and Lip@MOF decreased (Figure 6), indicative of the interaction of the polymer with the enzyme or possible increased diffusion limitations within the MOF biocatalyst. This highlights the favorable use of PAA as a MOF coating as it can increase stability while retaining biocatalytic activity. This concept could further be applied to other MOF systems.

## Conclusions

A critical aspect of enzyme@MOF studies has been illustrated, and emphasis has been placed on the importance of the stability of the MOF support. We have highlighted components of enzymatic assays that can have a profound effect on the stability of MOFs. This is an essential aspect to be studied in the use of MOFs for the encapsulation of enzymes. The buffer composition and concentration and MOF architecture and properties all play a role in the stability of the support and must be considered when evaluating the materials for use as supports for the immobilization of enzymes. The instability of MOFs in citrate buffer demonstrated that this buffer should be avoided. Acetate was identified as a promising substitute. However, use of citrate may provide scope for the on-demand release of enzymes or drugs from the MOF supports. Recently, Suresh and Matzger^66^ exploited hydrolytic degradation of the non-toxic MOF-5 in drug delivery systems for the release of drugs in aqueous environments. We have also identified that some buffers are compatible with the MOFs analyzed. For example, if acidic pH is required, acetate buffer should be used rather than citrate. Additionally, potassium phosphate and Tris–HCl generally allowed for good storage stability. However, Co-TMA and Ni-TMA were not stable in any of the buffers examined and should be avoided as supports for biocatalysis. The work presented provides an insight into MOF-buffer combinations that can be successfully utilized. These have been outlined in Table 1 and can provide a basis for the selection of a MOF support. A clear understanding of the hydrolytic stability of the MOF supports in buffer is understood and can allow for better use of the supports in future applications while also providing a stable environment. Additionally, the screening of polymers outlined that PAA can be used to increase MOF stability in aqueous solutions while retaining catalytic activity of the entrapped enzyme. This represents an interesting concept which can be explored further in MOF applications for biocatalytic mechanisms.

**Table 1:**
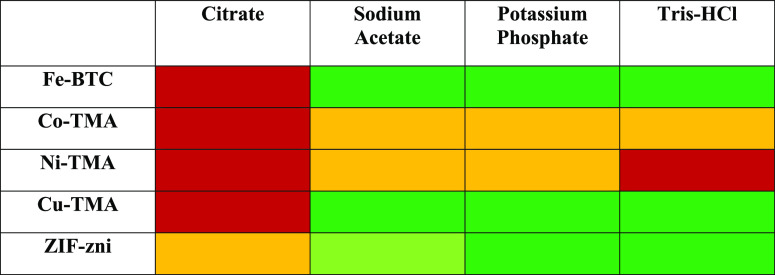
MOF–Buffer Combination Compatibility Rated by the Effect on MOF Stability^a^

a The color key is as follows, dark green: very good, light green: good, orange: fair, and red: poor.

## Supporting Information Available

Additional experimental details and data, MOF synthesis images, MOF metal content, calibration curves, SEM, SDS-PAGE, additional enzymatic activity assaying, polymer characteristics, and MOF–buffer–polymer storage images (PDF)

## Abbreviations

MOF: metal organic framework
PAA: polyacrylic acid
ADH: alcohol dehydrogenase
Lip: lipase
GOx: glucose oxidase
Lac: laccase
HEPES: 4-(2-hydroxyethyl)-1-piperazineethanesulfonic acid
Tris: 2-amino-2-hydroxymethyl-propane-1,3-diol
PBS: phosphate buffered saline
MES: 2-(*N*-morpholino)ethanesulfonic acid
ALDH_Tt_: aldehyde dehydrogenase from *Thermus thermophilus*
LDH: l-lactate dehydrogenase
NAD^+^: nicotinamide adenine dinucleotide
NADH: nicotinamide adenine dinucleotide hydrate
PEG: polyethylene glycol
SEM: scanning electron microscopy
ICP-OES: inductively coupled plasma optical emission spectroscopy
ZIF: zeolite imidazole framework
DMF: dimethylformamide
THF: tetrahydrofuran
PAC: (*N*,*N*′-bis(acryloyl)cytamide)
PEI: polyethyleimine
HRP: horse radish peroxidase
