# Supplementary material for: Enzyme Immobilization on Metal Organic Frameworks: the Effect of Buffer on the Stability of the Support
Source: Langmuir. 2022 Oct 26;38(44):13382–91. doi: 10.1021/acs.langmuir.2c01630 (PMC9648341; doi:10.1021/acs.langmuir.2c01630)
Supplement: Supplementary file 1 — la2c01630_si_001.pdf [file la2c01630_si_001.pdf]

Supporting Information

Enzyme Immobilization on Metal Organic Frameworks: The Effect of Buffer on the Stability of the Support

Kim Shortall, Fernando Otero, Simon Bendl, Tewfik Soulimane and Edmond Magner.*

Department of Chemical Sciences, Bernal Institute, University of Limerick, V94 T9PX, Ireland.

Corresponding Author

*Email: Edmond.magner@ul.ie

Table of Contents

Figure S1: Fe-BTC synthesis, A: solution 1 & 2 combined, B: solution 3, C: instant formation of a reddish-brown solid upon dropwise addition of $FeCl_3$ into trimesic acid solution, D: MOF suspension, E: filtering and drying. S2

Figure S2: Synthesis of Co-TMA (pink), Ni-TMA (green) and Cu-TMA (blue). A: MOF suspensions, B: dry MOFs. S3

Figure S3: ZIF-zni S3

Figure S4: Calibration curve of $FeCl_3$ at 295 nm. S4

Figure S5: Calibration curve of p-nitrophenol at 348 nm in 50 mM sodium phosphate pH 7. .....S4

Figure S6: SEM of MOFs. A & B: Co-TMA, C & D: Ni-TMA, E & F: Cu-TMA, G & H: ZIF-zni, I & J: Fe-BTC. S5

Table S1: Metal content of MOFs determined using ICP-OES..S6

Table S2: pH maintenance of Fe-BTC storage solutions after 48 h incubation in various buffers of different ionic strengths, pH 5 citrate, pH 7 potassium phosphate, pH 9 Tris-HCl S6

Figure S7: Detection of immobilization of $ALDH_{Tt}$ in Fe-BTC, lane 1: PageRuler prestained protein ladder (ThermoFisher Scientific), lane 2 & 3: $ALDH_{Tt}$@MOF samples, Fe-BTC immobilization supernatant...S6

Figure S8: In-situ immobilization of LDH in ZIF-zni and Cu-TMA demonstrating the biocatalysts relative specific activity compared to soluble LDH...S7

Table S3: Polymers used in Fe-BTC storage stability enhancement trials, highlighting their associated properties. S7

Figure S9 : Incorporation of polymers for increased stability of Fe-BTC. All samples are stored in 10 mM citrate pH 5 and photographed after 24 h, blank, 4% v/v and 8% v/v polymer samples are indicated. A: polyethylene glycol (PEG), B: polyethyleneimine (PEI), C: Tween polysorbate 20, D: polyacrylic acid (PAA)..S8

Figure S10: Activity of enzymes, A: $ALDH_{Tt}$, B: LDH and C: Lip in 4 and 8% PAA...........S9

Figure S11: Absorbance vs. time of Lip@MOF samples at 348 nm, using 0.4 mM p-NPA with the addition of varying concentrations of PAA. A: Lip@MOF, B: Lip@MOF 4% PAA, C: Lip@MOF 8% PAA. S9

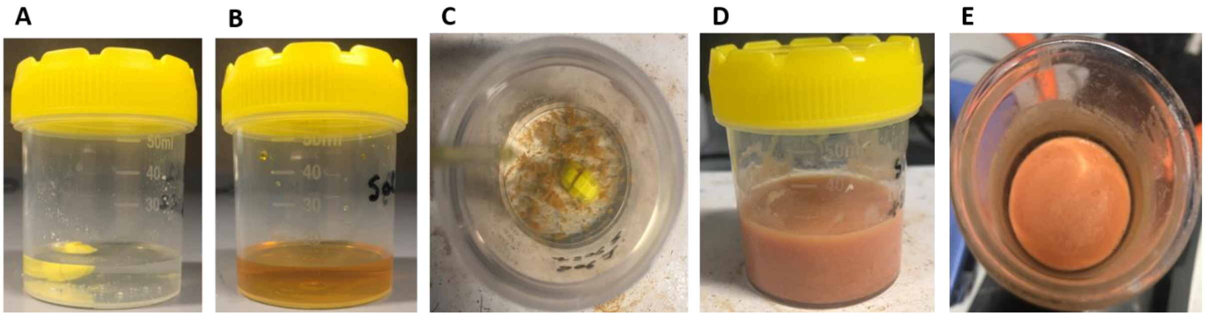

Figure S1: Fe-BTC synthesis, A: solution 1 & 2 combined, B: solution 3, C: instant formation of a reddish-brown solid upon dropwise addition of $FeCl_3$ into trimesic acid solution, D: MOF suspension, E: filtering and drying.

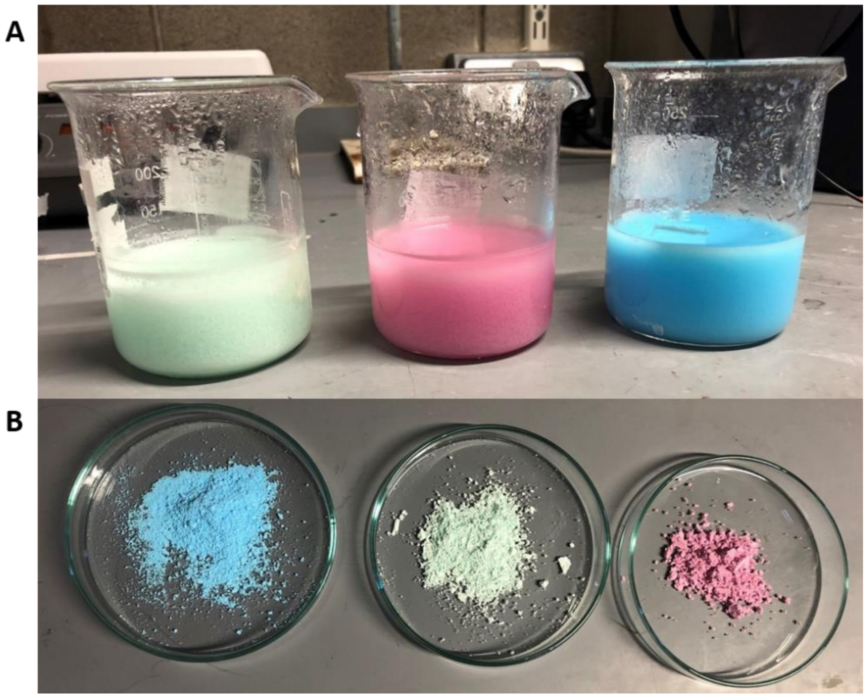

Figure S2: Synthesis of Co-TMA (pink), Ni-TMA (green) and Cu-TMA (blue). A: MOF suspensions, B: dry MOFs.

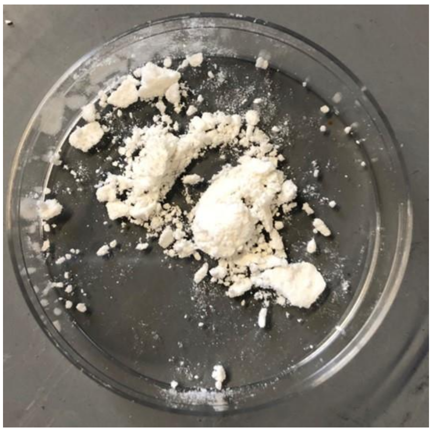

Figure S3: ZIF-zni

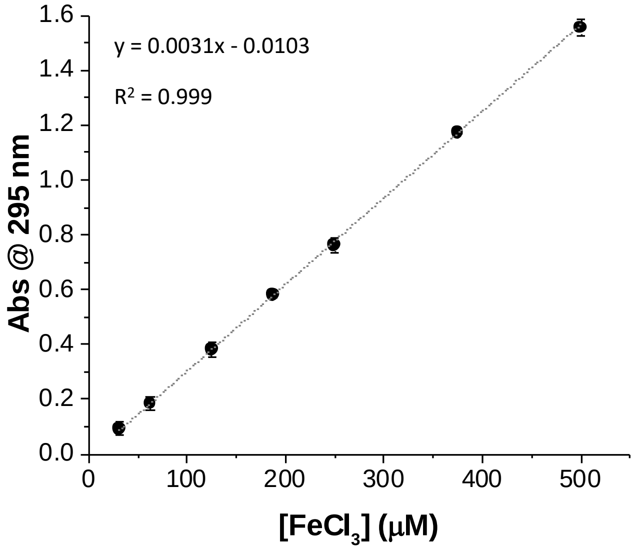

Figure S4: Calibration curve of $FeCl_3$ at 295 nm.

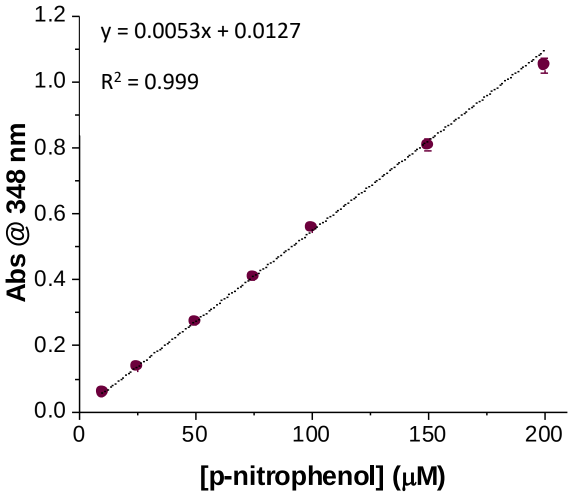

Figure S5: Calibration curve of *p*-nitrophenol at 348 nm in 50 mM sodium phosphate pH 7.

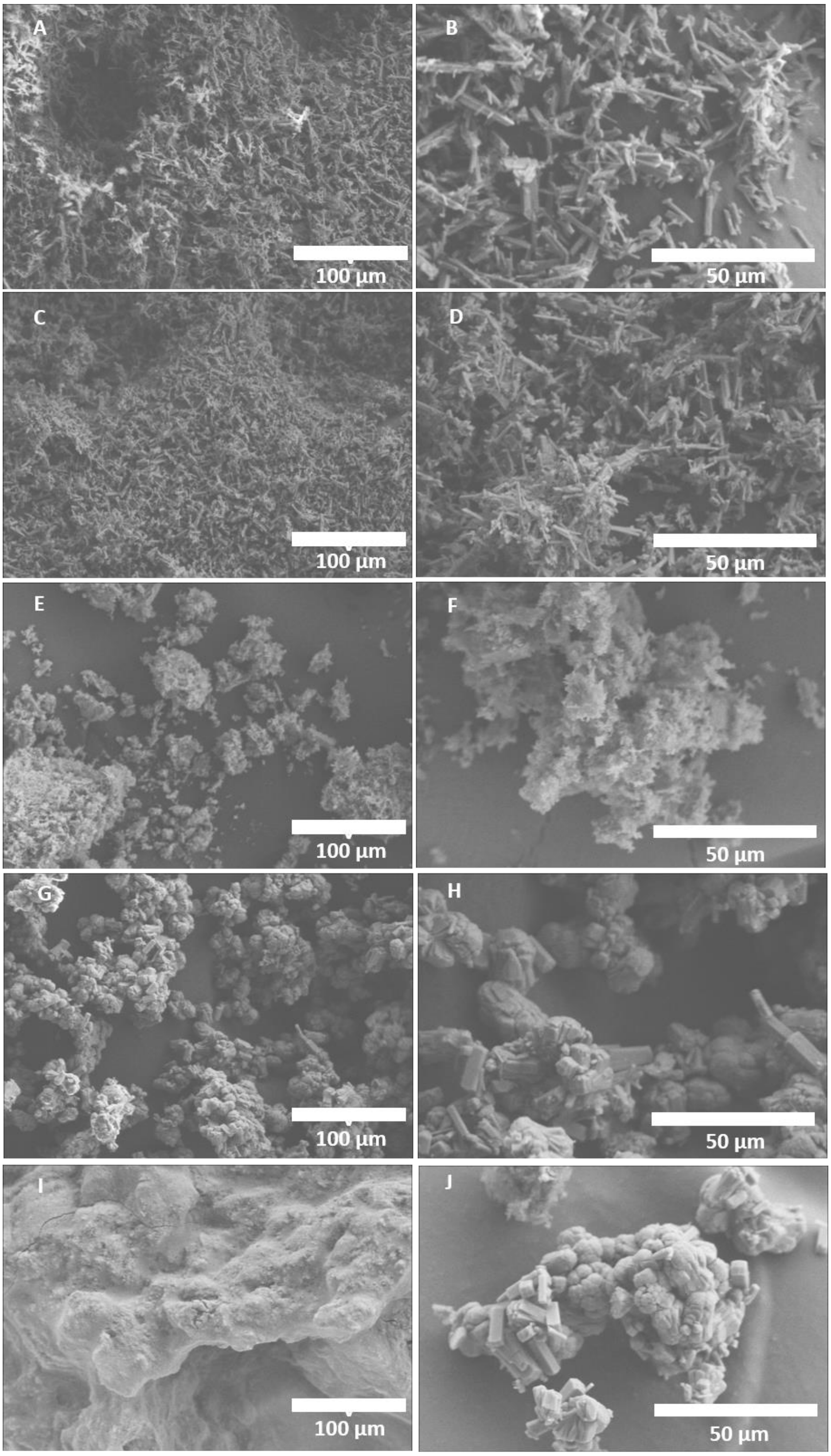

Figure S6: SEM of MOFs. A & B: Co-TMA, C & D: Ni-TMA, E & F: Cu-TMA, G & H: ZIF-zni, I & J: Fe-BTC.

MOF	Metal Content (mg/mg MOF)	Theoretical metal content (mass %)
**Fe-BTC**	0.034 ± 0.00026	4.2-8.1
**Co-TMA**	0.23 ± 0.0024	29.6
**Ni-TMA**	0.20 ± 0.0013	29.5
**Cu-TMA**	0.25 ± 0.0040	31.2
**ZIF-zni**	0.21 ± 0.023	32.4

Table S1: Metal content of MOFs determined using ICP-OES

	pH	5	7	9
**10 mM Buffer**	Static	4.3	6.5	7.8
	Agitated	3.9	5.0	5.3
**100 mM Buffer**	Static	4.9	6.9	8.5
	Agitated	4.9	6.9	8.6

Table S2: pH maintenance of Fe-BTC storage solutions after 48 h incubation in various buffers of different strengths, pH 5 citrate, pH 7 potassium phosphate, pH 9 Tris-HCl.

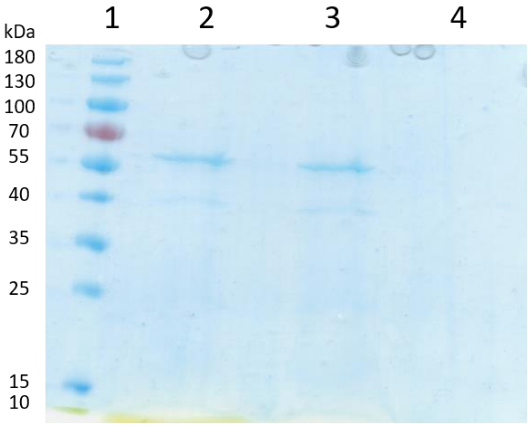

Figure S7: Detection of immobilisation of $ALDH_{Tt}$ in Fe-BTC, lane 1: PageRuler prestained protein ladder (ThermoFisher Scientific), lanes 2 & 3: $ALDH_{Tt}$@MOF samples, lane 4: Fe-BTC immobilization supernatant.

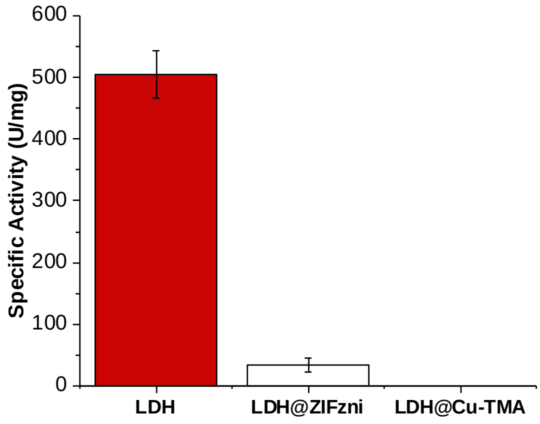

Figure S8: In-situ immobilisation of LDH in ZIF-zni and Cu-TMA demonstrating the biocatalysts relative specific activity compared to soluble LDH. Error bars refer to separate measurements.

	**Charge at pH 7**	**Acidity/ Basicity**	**Hydrophilicity**	**Application**
**Polyacrylic Acid**	+	Acidic	Hydrophilic	Used in drug delivery
**Polyethylene imine**	-	Basic	Hydrophilic	Drug delivery
**Polyethylene Glycol**	0	Neutral	Hydrophilic	Surfactant, shielding polmer, drug delivery
**Polysorbate 20, Tween**	0	Neutral	Amphiphilic	Non-ionic surfactant, solubilizer, additive in drug delivery

Table S3: Polymers used in Fe-BTC storage stability enhancement trials, highlighting their associated properties.

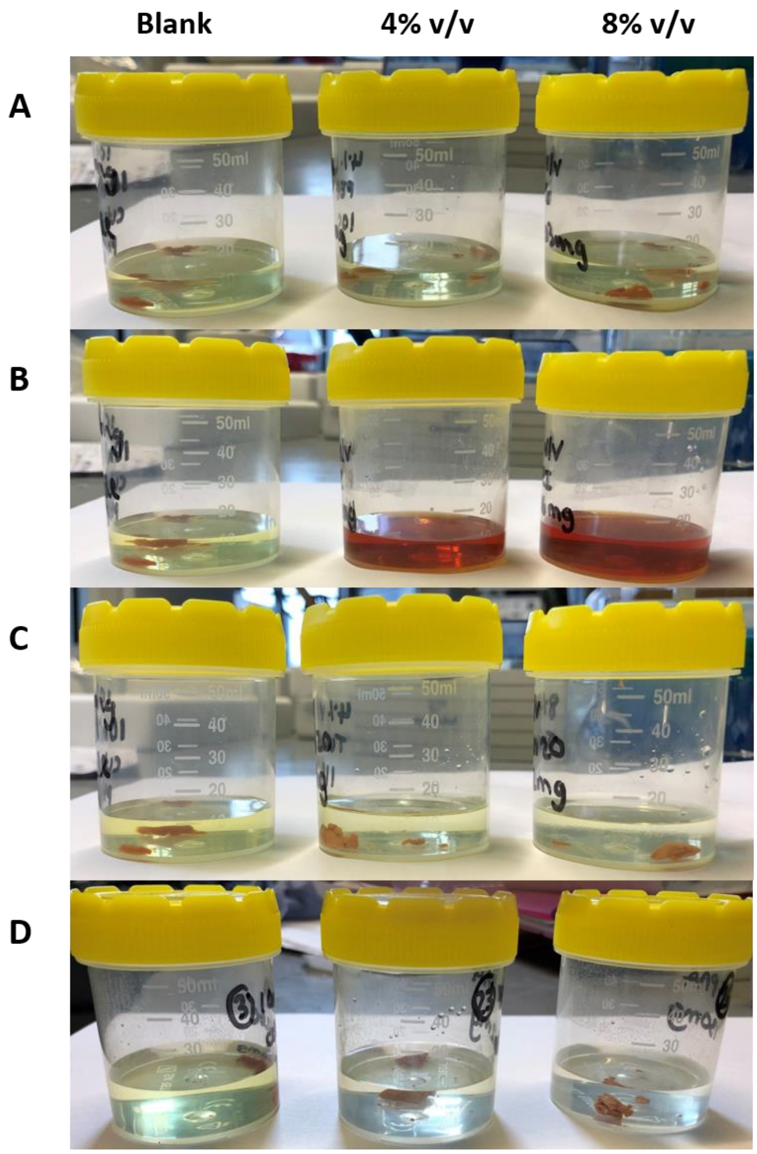

Figure S9: Incorporation of polymers for increased stability of Fe-BTC. All samples are stored in 10 mM citrate pH 5 and photographed after 24 h, blank, 4% v/v and 8% v/v polymer samples are indicated. A: polyethylene glycol (PEG), B: polyethyleneimine (PEI), C: Tween polysorbate 20, D: polyacrylic acid (PAA).

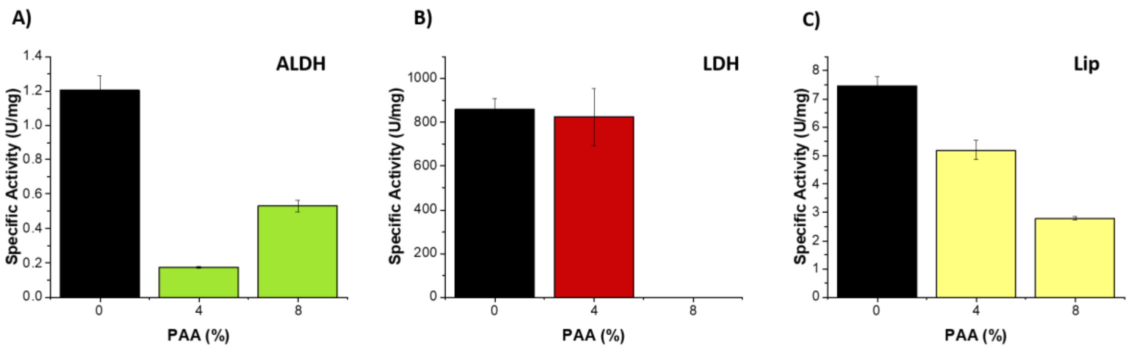

Figure S10: Activity of enzymes, A: $ALDH_{Tt}$, B: LDH and C: Lip in 4 and 8% PAA. Error bars refer to separate experiments.

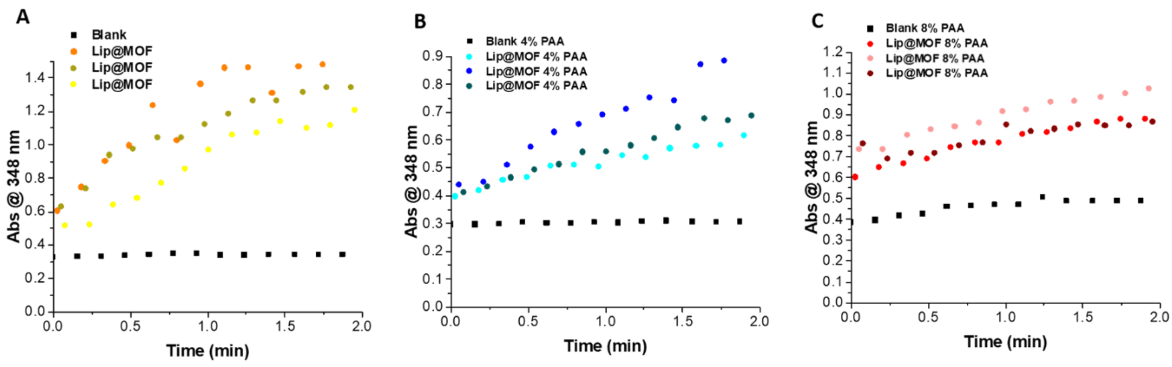

Figure S11: Absorbance vs. time of Lip@MOF samples at 348 nm, using 0.4 mM *p*-NPA with the addition of varying concentrations of PAA. A: Lip@MOF, B: Lip@MOF 4% PAA, C: Lip@MOF 8% PAA.
